# Spatially Correlated Nuclear Magnetic Resonance Profiles as a Tool for Precision Agriculture

**DOI:** 10.1021/acs.jafc.2c08265

**Published:** 2023-03-09

**Authors:** Raffaele Lamanna, Gerardo Baviello, Marcello Catellani

**Affiliations:** † Italian National Agency for New Technologies, Energy and Sustainable Economic Development (ENEA), Biotechnology and Agro-Industry Division, Trisaia Research Center, SS 106 Jonica Km 419.5, 75025 Rotondella, Matera, Italy; ‡ Italian National Agency for New Technologies, Energy and Sustainable Economic Development (ENEA), Biotechnology and Agro-Industry Division, Casaccia Research Center, Via Aguillarese 301, 00123 Rome, Italy

**Keywords:** NMR, precision agriculture, profiling, GIS, metabolic index

## Abstract

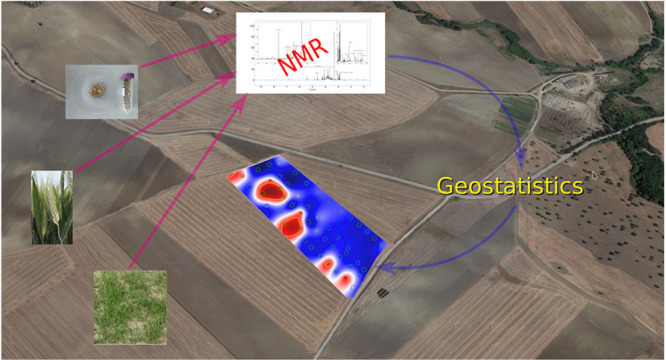

Nuclear magnetic resonance (NMR) profiling, sample georeferentiaton, and geostatistics are applied to evaluate the spatial variability of metabolic expression of durum wheat in fields managed by precision agriculture. Durum wheat at three different vegetation stages, grown in two different places of the Basilicata region, in Italy, is analyzed by NMR. The spatial variability, within each field, of metabolites, quantified by NMR, is evidenced by appropriate geostatistic tools through the definition of a suitable metabolic index. Metabolic maps are compared to highlight the effects of soil and farming strategies.

## Introduction

Pedoclimatic conditions strongly affect the metabolome of the plant, actually giving the imprint of the territorial origin to the chemical profile of many foods.

During the past 2 decades, a significant correlation between the metabolic content of a food and its geographic origin has been established by chemical profiling and, in particular, nuclear magnetic resonance (NMR). Many studies involving several foods and locations have been published using both targeted and untargeted NMR analysis assisted by multivariate statistics.^1−10^ In those studies, the relation between the metabolome and territorial origin were settled, by appropriate machine learning algorithms, according to the concentration of a set of metabolites. However, geographic information is in the process only marginally through the *a priori* definition of the territorial classes. These classes are quite arbitrarily identified according to administrative borders, cultural regions, hydrogeological networks, roads or railways, or statistical aggregation,^11^ and geographic differences inside those regions are simply ignored. Unfortunately, the class boundaries, thus defined, may or may not correspond to the positions of changes of the target variables.

The identification of territorial origin by multivariate statistical analysis of metabolic profiles is actually based on the hypothesis that geographic classes are spatially uncorrelated, while samples within a class are fully correlated.

This hypothesis is sustained by the observation that very far locations had different pedoclimatic histories that influenced the metabolic expression of plants in an uncorrelated way. On the other hand, we expect that the average pedoclimatic conditions of nearby locations belonging to the same class are very similar, making the metabolic profile of plants grown in that region strongly correlated. The long distance among the classes, with respect to interclass dimensions, is then crucial to disentangle pedoclimatic oscillations. With these assumptions, we expect that the differences of mean metabolic profiles among the classes are greater than the varibility within each class. These differences, when really present in the metabolic profiles of the analyzed foods, are evidenced by multivatiate statistical analysis.

There are however cases in which the above-mentioned conditions are not completely fulfilled.

In particular, when the spacing between classes is comparable to the distances among interclass samples, pedoclimatic oscillations cannot be disentangled by distance and samples belonging to different regions may have felt similar conditions; thus, the multivariate appoach might no longer be suitable, and the spatial sample correlation has to be taken into account. Actually, in this case, the possibility that target variable spatial changes might not coincide with the *a priori* identified class boundaries becomes a serious source of error.

Georeferentiation of the sample, that is the assignment of coordinates to each sample, and geostatistics may be used to identify and solve this issue.

Actually, in a previuos paper, we have used georeferenced olive oil samples and geostatistics to identify, according to spatial variability of NMR profiles, the boundaries of homogeneous quality adjacent regions, which can be used next as geographic classes in successive multivariate statistical approaches.^12^

When dimensions are smaller, at level of a single field, then spatial inhomogeneities are dominated by pedologic conditions, while climate effects mainly influence temporal behavior. Actually, the variability of both soils and climatic conditions involves different spatial and temporal scales.

Precision farming exploits the knowledge of soil spatial variability to adapt agromonic pactices to provide water and nutrients in those field areas where they are needed more, with obvious advantages in terms of resource savings and improved productivity.

Monitoring the effects of soil variability and the precision farming technique on the metabolic expression of plants in different field positions is extremely important because the soil structure and composition change within and among the fields. In fact, several factors contribute to soil spatial variability: parental material relief, organisms, climate, time, previous managements, and crops.

To evaluate soil spatial variability, several approaches are used combining proximal and remote sensing. Apparent electrical conductivity, vegetation indices by reflectance of radiation in several spectral bands, and other properties, such as crop yields and soil properties and composition, are some of the variables considered to assess field spatial variability.

However, to our knowledge, a plant metabolic profiling approach has never been attempted.

In this work, we analized by NMR profiling the metabolic expression of durum wheat at three different vegetation stages and in two different fields of the Basilicata region in the south of Italy conducted by precision farming techniques.^13^ The use of georeferenced samples and geostatistics allowed for the evaluation of the spatial variability of the metabolome at each development stage.

## Materials and Methods

### Sampling

Samples of main and secondary wheat shoots (13 weeks from sowing) and fully ripe ears were collected in a Genzano di Lucania (Potenza) field (latitude, 40.82° N; longitude, 16.08° E) in 2019, while spikes in full bloom and ripe ears were collected in a field near Matera (latitude, 40.71° N; longitude, 16.65° E) in 2020.

**Figure 1:**
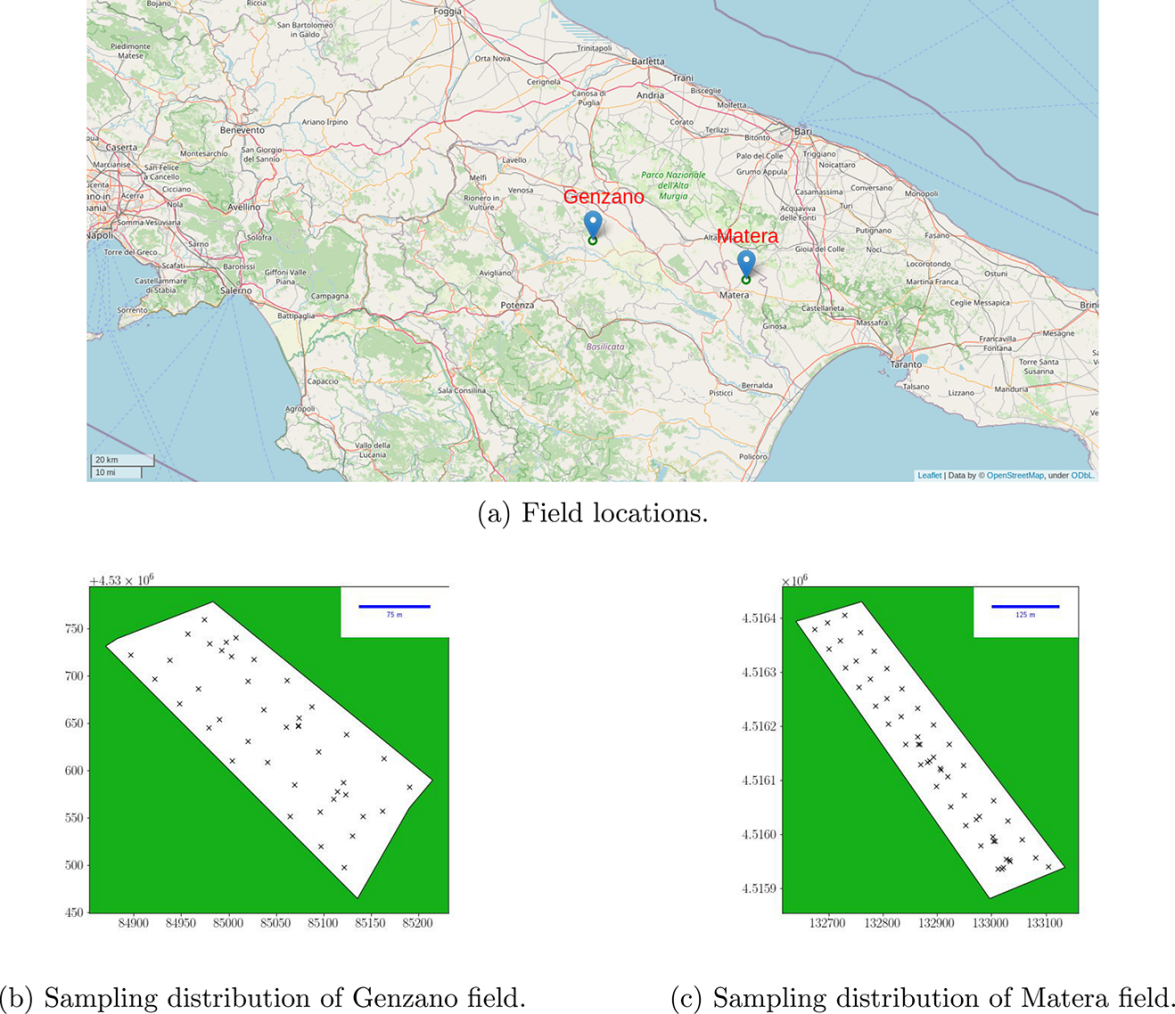
Analyzed field positions and an example of sampling distributions.

A few shoots or ears were collected from a region approximately 2 m wide in different georeferenced positions of the fields (panels b and c of Figure 1) and freezed at −20 °C within a few hours.

Wheat shoots were lyophilized and stored in dark and dry conditions until analysis.

Data collection was georeferenced by the Global Positioning System (GPS, Garmin GPSMAP 64S) with an error of 3 m.

Both fields were characterized and conducted by the University of Basilicata, in the framework of the same research project, according to a precision farming protocol. Cultivation details and characterization of both fields are described in detail by Denora et al.^13^

### Sample Preparation

Lyophilized green material (shoots and flowering ears) were ground in an IKA ULTRA-TURRAX homogenizer with 20 steal balls at 4000 rpm for 2 min. About 30 mg of powder was then dissolved into 1.4 mL of a mixture of 50% (v/v) CDCl_3_ and 180 mM D_2_O phosphate buffer at pH 6.8. The buffer was prepared by dissolving 1.508 g of sodium phosphate monobasic dihydrate (NaH_2_PO_4_·2H_2_O) and 1.180 g of sodium phosphate dibasic (Na_2_HPO_4_) in 100 mL of D_2_O.

After threshing of the ripe ears by a laboratory machine, grain was ground into a micro mill (Molina, Komo). Wheat flour was extracted in D_2_O phosphate buffer a pH 6.8. About 30 mg of wheat flour was dissolved in 700 μL of D_2_O phosphate buffer, vortexed for 10 min, and then shaken at 450 rpm for 10 min. After centrifugation at 12000*g* for 10 min, 500 μL of supernatant was transferred into a 5 mm outer diameter NMR tube with 50 μL of a 1 mM D_2_O solution of 2,2-dimethyl-2-silapentane-5-sulfonic acid (DSS) and 25 μL of a D_2_O solution of 80 mM NaN_3_.

Deuterated chloroform (99.96%) was purchased by Merk, while deuterium oxide (99.96%) came from Cambridge Isotope Laboratories, Inc.

### NMR

^1^H NMR spectra were acquired on a Bruker 600 Avance spectrometer at a proton frequency of 600.13 MHz and temperature of 298 K, with a 45° pulse of 5.63 μs, relaxation delay of 2 s, and 1024 scans. The strong residual HOD signal was suppressed by presaturation during the relaxation delay. Each free induction decay (FID) was Fourier-transformed with exponential apodization corresponding to a 0.3 Hz line width. Spectra were phase- and baseline-corrected and calibrated to the signal of DSS at 0.015 ppm.

Peak annotation was performed by literature data,^14^ and ambiguous assignments were confirmed by correlation spectroscopy (COSY), heteronuclear single quantum correlation (HSQC), and *J*-resolved spectroscopy (JRES).

**Figure 2:**
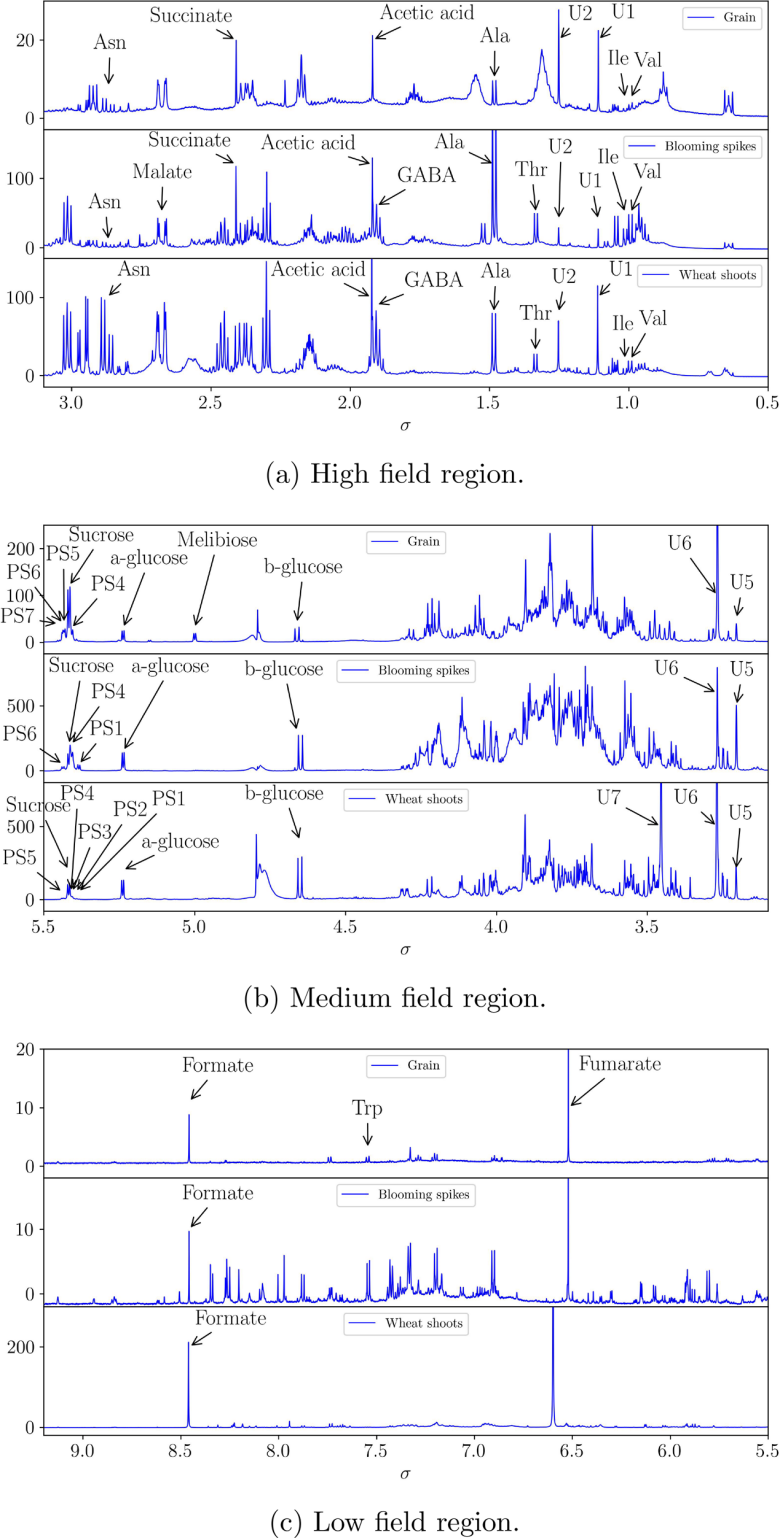
^1^H NMR spectra of wheat in the three vegetation stages.

Representative spectra with peak annotation are reported in Figure 2.

Metabolite quantification was made by resonance deconvolution with an appropriate line shape, as summarized in Table 1, where quantified compounds, the chemical shift of the corresponding deconvoluted resonance, and the line shape used are reported for the three vegetation stages analyzed.

**Table 1:** Quantified Signals

(a) wheat shoots of Genzano			(b) blooming spikes of Matera		
compound	σ	line shape	compound	σ	line shape
Val	0.9958	d	Val	0.9946	d
Ile	1.0139	d	Ile	1.0132	d
U1	1.1119	s	U1	1.1100	s
U2	1.2528	s	U2	1.2515	s
Thr	1.3356	d	Thr	1.3335	d
Ala	1.4850	d	Ala	1.4828	d
GABA	1.9073	5m	GABA	1.9073	5m
acetic acid	1.9230	s	acetic acid	1.9202	s
Asn	2.8734	dd	succinate	2.4090	s
U5	3.2063	s	malate	2.6770	dd
U6	3.2694	s	Asn	2.8682	dd
U7	3.4551	s	U5	3.2052	s
b-glucose	4.6511	d	U6	3.2683	s
a-glucose	5.2388	d	b-glucose	4.6613	d
PS1	5.3860	d	a-glucose	5.2373	d
PS2	5.3944	d	PS1	5.3840	d
PS3	5.4064	d	PS4	5.4094	d
PS4	5.4100	d	sucrose	5.4168	d
sucrose	5.4181	d	PS6	5.4367	d
PS5	5.4378	d	formate	8.4584	s
formate	8.4586	s			

(c) wheat flour				
compound	σ	line shape	(d) abbreviations	
Val	0.9946	d	Val	valine
Ile	1.0132	d	Ile	isoleucine
U1	1.1100	s	U	unassigned
U2	1.2515	s	Thr	threonine
Ala	1.4828	d	Ala	alanine
acetic acid	1.9202	s	GABA	γ-aminobutyric acid
succinate	2.4090	s	Asn	asparagine
Asn	2.8682	dd	a-glucose	α-glucose
U5	3.2052	s	b-glucose	β-glucose
U6	3.2683	s	PS	polysaccharide
b-glucose	4.6613	d	Trp	tryptophan
melibiose	5.0000	d	s	Lorentzian
a-glucose	5.2373	d	d	doublet
PS4	5.4094	d	dd	double doublet
sucrose	5.4168	d	5m	quintuplet
PS5	5.4332	d		
PS6	5.4367	d		
PS7	5.4510	d		
fumarate	6.5200	s		
Trp	7.5452	d		
formate	8.4584	s		

Spectra processing [fast Fourier transform (FFT) and phase correction] was made by Bruker Topspin version 3.6 software, while baseline correction, annotation, and line deconvolution where accomplished by the tNMR program.^15^ Data were then stored into a HDF5 database file containing line integrals and annotations together with some metadata, such as the sample geographic information system (GIS) coordinates.

### Statistical Analysis

Statistical analysis was performed in a Python script. Data read from the HDF5 files were transformed into a GeoPandas^16^ data frame. Sample positions detected in degrees were transformed to Universal Transverse Mercator coordinates in meters (WGS84/UTM zone 34N; datum, WGS84; EPSG, 32634) before analysis.

### Metabolic Index Calculation

The mean metabolic indexes MI_mean_ were evaluated by averaging all metabolite concentrations, after standard scaling, at each field position by the GeoPandas built-in function.

The coefficient of variation (CoV) of MI was calculated according to the following equation:
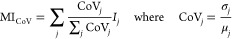
1with *j* spanning over all quantified metabolites and μ_*j*_ and σ_*j*_ being the mean and standard deviation of the *j*th metabolite over all of the sampling positions.

For MI_PCA_, the first principal component was considered. Principal component analysis (PCA) was calculated by the scikit-learn^17^ Python library in a pipline in which both standard data scaling and PCA were estimated in a single process.

### Geostatistics

Moran’s index was estimated by the PySal library^18^ moran(...) function with spatial weights based on the kernel triangular function, with Euclideian distance and nearest point bandwidth with *k* = 7 [weights.distance.Kernel.from_dataframe(...) method].

Histograms with kernel distribution functions were produced by the Seaborn Python library^19^ [histplot(...) and kdeplot(...), respectively].

Quantile–quantile (Q–Q) plots were made by the qqplot(...) method of the statsmodels library.^20^

Statistical interpolation was accomplished in the GSTools library^21^ by ordinary kriging [krige.Ordinary(...)] with an exponential model of the data-estimated variagram [vario_estimate(...)] with 20 standard bins.

Deterministic interpolation was performed by the radial basis function in the SciPy library.^22^

## Results and Discussion

From NMR spectra of plant aqueous extracts, different metabolites have been identified and quantified in each of the three analyzed vegetation stages, as summarized in Table 1.

Each plant stage had a peculiar metabolic profile, as shown by NMR spectra (Figure 2) and metabolite mean values reported in Figure s1 of the Supporting Information.

In this work, we are mainly interested in the spatial distribution of the metabolic content that is in the variation of the metabolome among the different field positions.

To understand the metabolic variability within each field, some statistics (mean, standard deviation, histogram, Q–Q plot, semivariogram, and spatial interpolation) have been estimated for each quantified metabolite.

**Figure 3:**
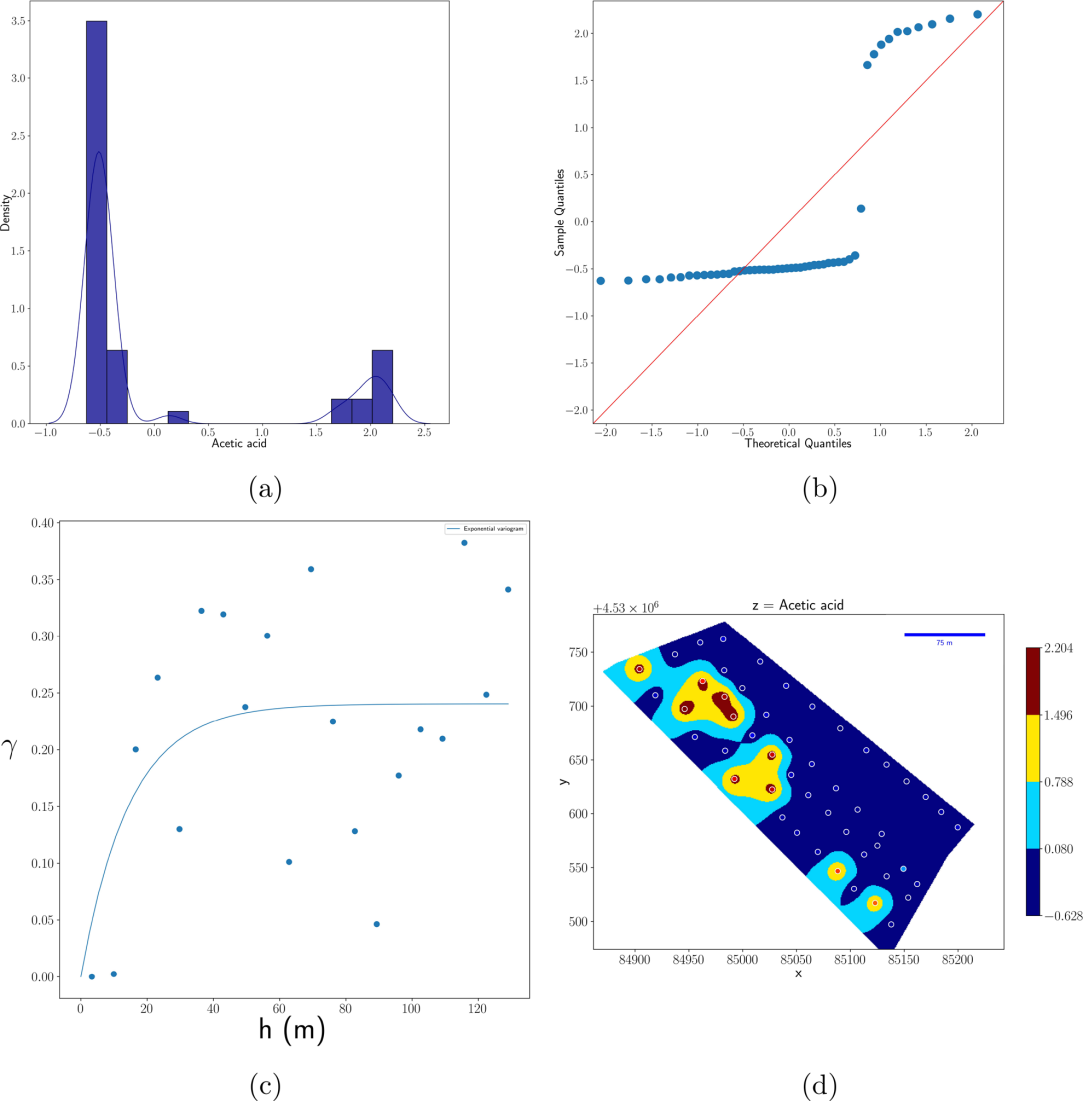
(a) Histogram, (b) Q–Q plot, (c) semivariogram, and (d) heat map of interpolated values by kriging of acetic acid. The variable was previously standardized. Dots in panel d represent, in a false color scale, the experimental values at the sampling positions. The red line in panel b represents the normal distribution.

**Figure 4:**
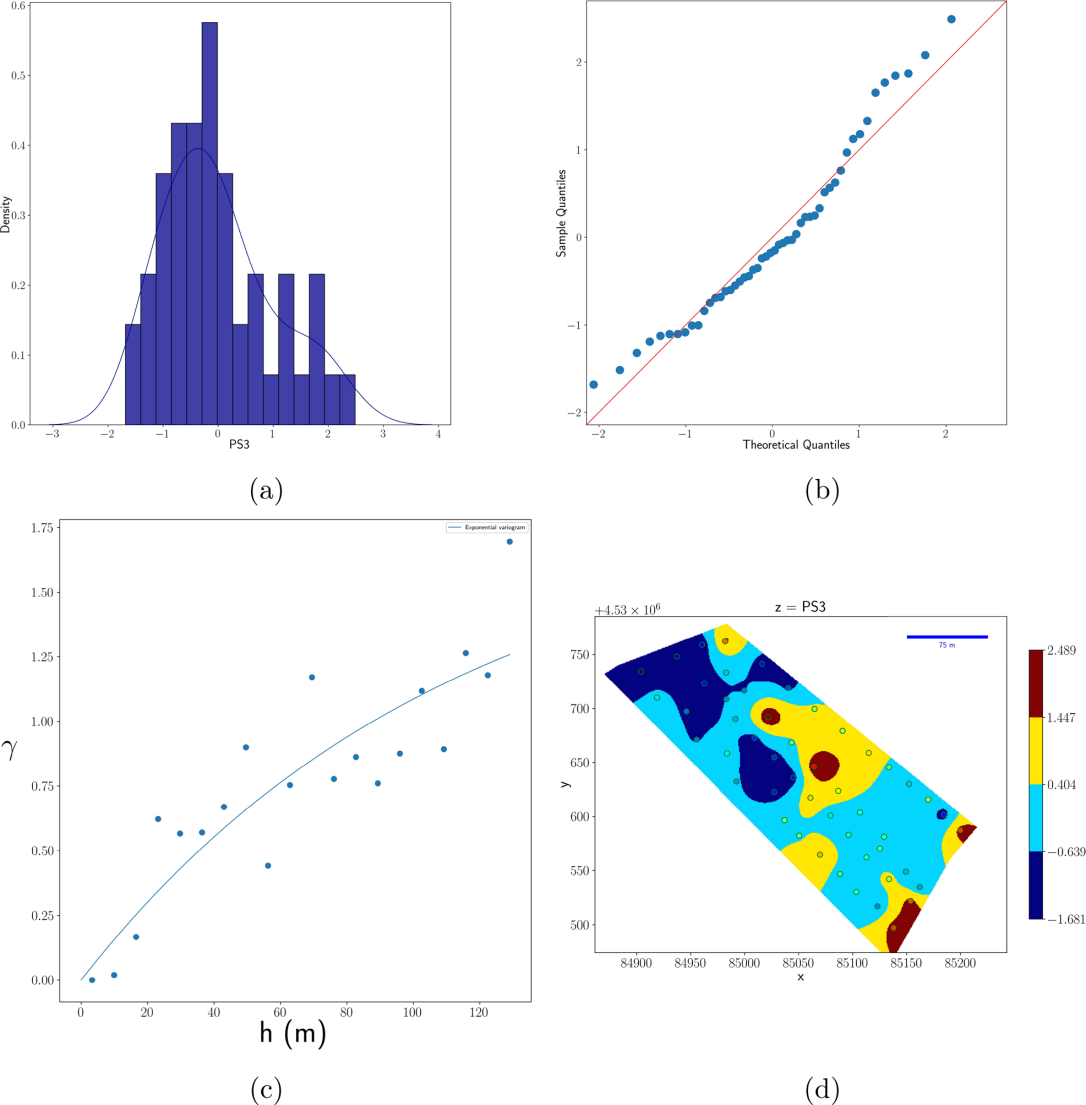
(a) Histogram, (b) Q–Q plot, (c) semivariogram, and (d) heat map of interpolated values by kriging of PS3. The variable was previously standardized. Dots in panel d represent, in a false color scale, the experimental values at the sampling positions. The red line in panel b represents the normal distribution.

As an example, in Figures 3 and 4, the histogram, quantile plot, semivariogram, and kriging interpolation map are reported, respectively, for two significant metabolites, quantified from extracts of main and secondary wheat shoots, namely, acetic acid and unknown polysaccharide PS3, being chosed for having the shortest and longest correlation lengths.

In Figures 3d and 4d, dots represent the value of experimental data in the position where samples were collected. The NMR-estimated concentration of the corresponding metabolite is represented by a color map whose scale is reported in the color bar. Kriging interpolation was used to determine the color of the map points between the experimental points. Kriging, which belongs to the statistical interplation class, actually exploits the information relative to the spatial correlation given by the semivariogram (Figures 3c and 4c) to estimate the value of a variable between experimental points.^12^ Also, deterministic interpolation algoritms are available, and an example based on radial basis functions is reported in Figure s2 of the Supporting Information for the two metabolites.

In the case of acetic acid, the distribution (Figure 3a) is markedly bimodal, with values centered at about −0.5 and 2.0, with the left peak containing more samples than the other peak.

As shown in Figure 3d, the samples relative to the left peak are strongly spatially correlated and mainly clustered in the large blue region. On the other hand, the points belonging to the right peak are grouped in smaller regions, as also evidenced by the semivarigram in Figure 3c, where the correlation length is nearly 15 m. The Q–Q plot has the typical sigma shape of a bimodal distribution.

In the case of PS3, bimodality is less evident. The two peaks are evidenced only by distribution estimation and are large and convoluted. Also, in this case, the left peak comprised the major part of the samples, as also evidenced by the prevalence of blue tones in Figure 3d. The patches of different colors are greater than those of acetic acid, in agrement with the semivariogram, which indicates a correlation length of 112 m. The Q–Q plot shows a close to Gaussian behavior, except for small deviations from the red line as a result of the slightly bimodal character of the data.

The behavior of the two compounds, taken as an example, is really different (Figures 3 and 4) and this is also the case for all of the other quantified metabolites (data not shown) as, in turn, indicated by the significat correlation length variability among the metabolites (see Tables s1–s4 of the Supporting Information).

It is evident that a single metabolite does not represent the overall metabolic field variability. Actually, considering the different behaviors of the quantified metabolites, the question arises if some spatial patterns should persist when the entire metabolome spatial variability is considered.

To answer this question, a global variable or, actually, a metabiolic index (MI) needs to be defined for summarizing all of the metabolic information.

A linear combination of the metabolite concentrations, MI = *∑*_*j*_*w*_*j*_*I*_*j*_, is the simplest choice for a metabolic index. However, different coefficient options will produce distinct alternative MIs. In Table 2, three possible MI definitions are reported.^23^ In particular, the mean calculation gives all of the variables the same relevance, while the coefficient of variation emphasizes the variables with a high variability. Finally, the first principal component will emphasize the variance among samples.

**Table 2:** Algorithms for Metabolic Index Calculation

MI	*w*_*j*_	
mean	1/*N*	all variables have the same relevance
CoV	CoV_*j*_/∑_*j*_CoV_*j*_ with CoV = σ/μ	gives importance to variables with high variability
PCA	PC1	emphasizes variance among sampling points

Naturally, before MI calculation, an appropriate variable scaling is needed.

Because each of the quantified metabolites have a different content of spatial variability, as shown by Moran’s index reported in Figure s3 of the Supporting Information, in principle, the sum-defining MI can be resticted only to those variables with a high geographic content.^12^ Actually, from Moran’s index, it appears that all of the analyzed variables have a significant spatial correlation. Thus, as a result of the difficulty of defining a suitable threshold for the geographic content, all of the quantified metabolites were used to calculate the MI by PCA.

**Figure 5:**
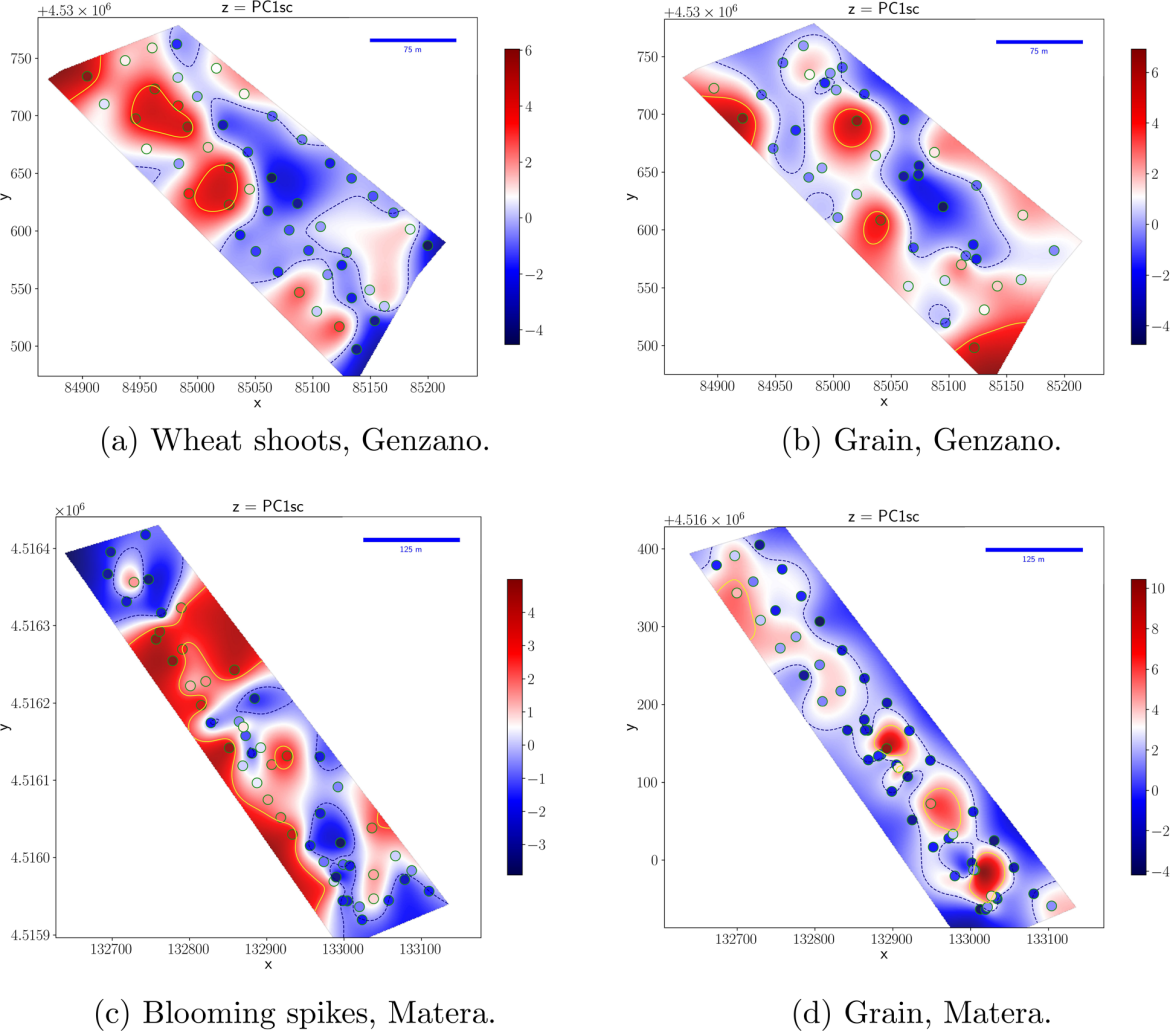
Deterministic interpolation by radial basis functions.

Figure 5 shows the maps of the PCA metabolic index for the different plant growth stages and locations (maps of MI_mean_ and MI_CoV_ are shown in Figures s4 and s5 of the Supporting Information, respectively). Each map is the deterministic interpolation, by radial basis functions, of the experimental data represented in the figure by dots.

The wheat shoot MI for the Genzano field, reported in Figure 5a, shows a significant spatial correlation, with positive values clustered in the northwest region and negative values grouped in the southeast region. On the other hand, the MI of grain produced in the same field (Figure 5b) displays a more random distribution among the different field locations, probably as a result of precision agriculture practices perfomed on the field or climatic conditions, which may level field characteristics. The spatial correlation seen in the early plant stage and the quite random distribution of the MI values in the grain metabolome are confirmed by Local Indicators of Spatial Association (LISA) analysis^24^ reported in Figure s6 of the Supporting Information.

A similar behavior is observable in the Matera field, where again spatial correlation is evident in the blooming spike metabolome (Figure 5c) contrary to the grain (Figure 5d), where a more uniform distribution is observable, except for central part of the field, where uniform fertilization was applied. However, from LISA analysis, it emerges that the spatial correlation in the Matera grain map is not significant and the uniform fertilization zone cannot be significantly identified by the map. The spatial distribution is so uncorrelated that the variogram is completely flat and kriging interpolation is impossible. That is why in Figure 4 deterministic interpolation was preferred over kriging.

**Figure 6:**
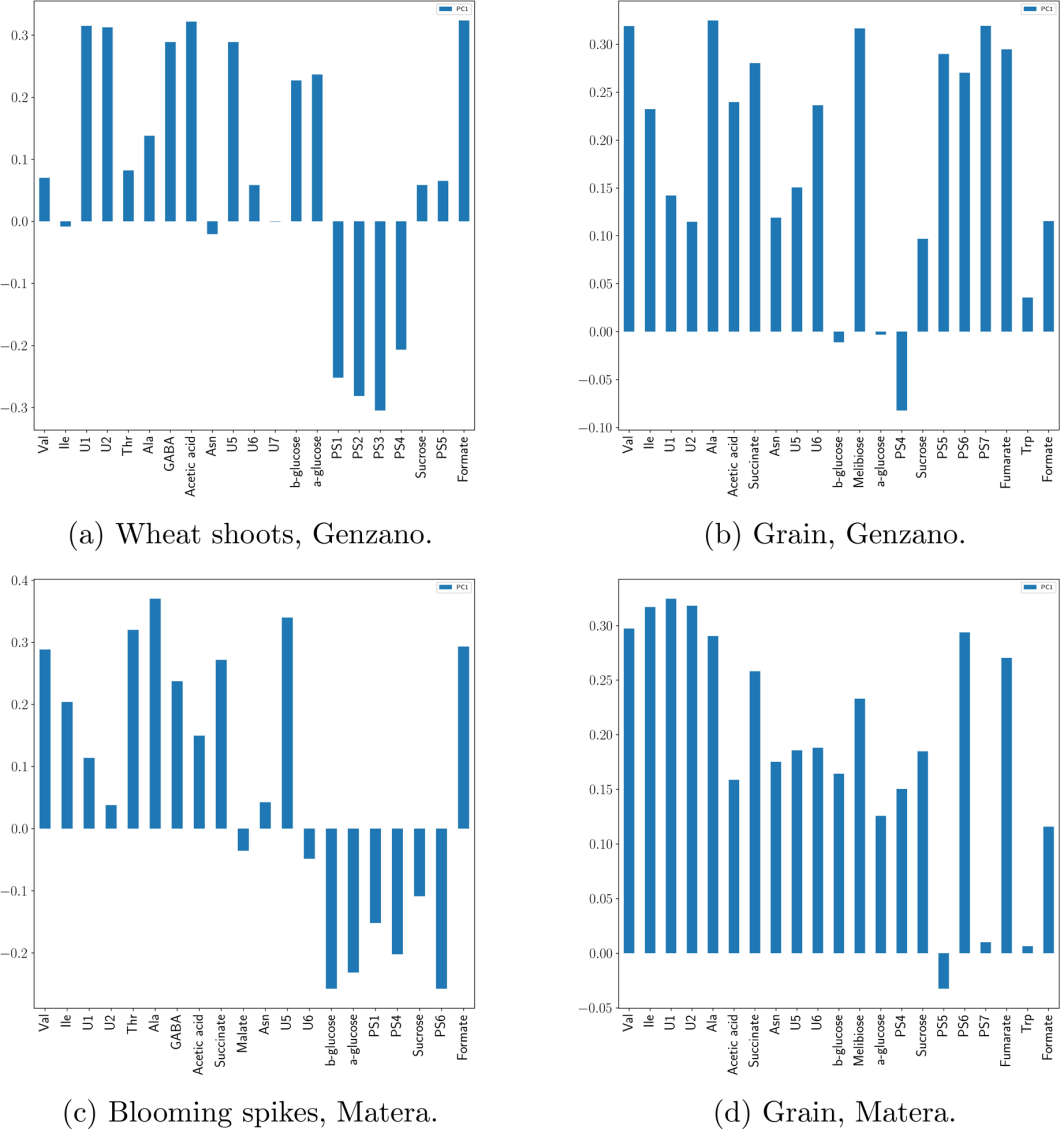
PC1 loadings. Positive loadings are positively correlated to red regions in the maps of Figure 4 and negatively correlated to blue regions. The reverse occurs for negative loadings.

PC1 loadings, which are related to the weights used to calculate the MI, are shown in Figure 6.

From Figure 6a, it appears that Val, U1, U2, Thr, Ala, GABA, acetate, U5, U6, β-glucose, α-glucose, sucrose, PS5, and formate are overexpressed in the northwest region (in red in Figure 5), while the southeast region (blue in Figure 5) is characterized by a greater concentration of polysaccarides (PS1, PS2, PS3, and PS4). A similar behavior is observed for blooming spikes in the Matera field where the red regions in the map correspond to a higher concentration of Val, Ile, U1, Thr, Ala, GABA, succinate, U5, and formate, while the map blue regions are characterized by β-glucose, α-glucose, PS1, PS4, sucrose, and PS6. On the other hand, the loadings of wheat flour of both fields show that the major part of the metabolites is positively correlated to MI.

In summary, metabolic profiling is demonstrated as an additional valuable tool to analyze spatial variability of farm land. Sample georeferentiation, geostatistics, and NMR profiling permit establishment of a relation between the metabolic expression of plants, in particular, durum wheat, and morphological inhomogeneities of agricultural fields. In addition, any external forcing on plant biology, such as climate events, pathogen infections, farming practices, etc., can be monitored in space and time, becoming a valuable help to precision farming strategies. This approach, despite not being at the moment applicable to routine field characterization, is important to validate other euristic and more fast field characterization tools, making a direct connection between spatial variability of soils and plant metabolic expression and eventually crop quality. In addition, a future comparison of metabolic profile maps to soil electrical conductivity and remote sensing spectroscopic results may strongly improve the understanding and use of such tools. In fact, a visual comparison of Figure 5 and Figure 3 of Denora et al.^13^ is encouraging, showing an evident similarity between MI and electrical conduntivity maps. Work is in progress to mathematically compare the two results.

## Supporting Information Available

Semivariogram fitting results, mean content of each quantified metabolite, radial basis function (RBF) interpolation of acetic acid and PS3, Moran’s index, map representation of mean and CoV metabolic indices, and LISA maps (PDF)
