# Supplementary material for: Spatially Correlated Nuclear Magnetic Resonance Profiles as a Tool for Precision Agriculture
Source: J Agric Food Chem. 2023 Mar 9;71(11):4745–54. doi: 10.1021/acs.jafc.2c08265 (PMC10037328; doi:10.1021/acs.jafc.2c08265)
Supplement: Supplementary file 1 — jf2c08265_si_001.pdf [file jf2c08265_si_001.pdf]

Spatially correlated NMR profiles as a tool for precision agriculture. Supporting Information

Raffaele Lamanna,*,† Gerardo Baviello,† and Marcello Catellani‡

†*ENEA, Italian National Agency for New Technologies, Energy and Sustainable Economic Development. Biotechnology and Agro-Industry Division, Trisaia Research Center, SS 106 Jonica Km 419.5, 75025 Rotondella (Mt), Italy*

‡ *ENEA, Italian National Agency for New Technologies, Energy and Sustainable Economic Development. Biotechnology and Agro-Industry Division, Casaccia Research Center, Rome, Italy*

E-mail: raffaele.lamanna@enea.it

Geostatistics

Semivarigram parameters

Tables s1 to s4 show the fitting parameters for an exponential regression of semivariogram for each of the quantified metabolites in the two analyzed fields.

Table s1: Genzano wheat shoots: Variogram fitting results

var	len_scale	nugget	Metabolite
0.91	24.86	0	Val
0.90	30.75	0	Ile
0.16	16.99	0	U1
0.21	16.45	0	U2
1.12	29.54	0	Thr
0.92	19.47	0	Ala
0.87	6.34	0	GABA
0.24	14.61	0	Acetic acid
1.11	37.25	0	Asn
1.03	28.04	0	U5
0.89	4.47	0	U6
0.80	32.76	0	U7
1.26	31.35	0	b-glucose
1.32	33.83	0	a-glucose
1.09	46.49	0	PS1
1.35	55.11	0	PS2
1.84	111.82	0	PS3
1.01	22.21	0	PS4
1.12	43.18	0	Sucrose
1.00	18.96	0	PS5
0.22	16.63	0	Formate
1.16	13.12	0	PC1
6.13	28.09	0	PC1sc
0.01	20.74	0	MiCoV
0.15	17.61	0	mean

Table s2: Genzano grain: Variogram fitting results

var	len_scale	nugget	Metabolite
1.00	10.11	0	Val
1.01	8.47	0	Ile
1.08	10.88	0	U1
1.04	17.33	0	U2
0.75	15.45	0	Ala
0.94	23.07	0	Acetic acid
1.08	17.46	0	Succinate
1.04	4.94	0	Asn
0.98	5.77	0	U5
0.96	17.80	0	U6
1.06	4.07	0	b-glucose
1.03	4.35	0	Melibiose
1.01	4.14	0	a-glucose
1.18	17.74	0	PS4
1.06	11.97	0	Sucrose
1.11	4.96	0	PS5
1.18	52.63	0	PS6
1.11	35.31	0	PS7
1.06	11.97	0	Fumarate
1.29	42.29	0	Trp
1.00	4.39	0	Formate
0.01	10.22	0	PC1
6.39	73.87	0	PC1sc
0.00	14.72	0	MiCoV
0.26	15.60	0	mean

Table s3: Matera blooming spikes: Variogram fitting results

var	len_scale	nugget	Metabolite
1.11	51.56	0	Val
1.00	31.54	0	Ile
0.70	3.05	0	U1
0.76	7.34	0	U2
0.89	24.35	0	Thr
1.40	60.36	0	Ala
0.67	12.15	0	GABA
1.39	79.60	0	Acetic acid
0.86	30.58	0	Succinate
0.99	15.56	0	Malate
0.74	3.66	0	Asn
1.06	8.09	0	U5
0.91	4.31	0	U6
1.31	59.49	0	b-glucose
1.29	47.52	0	a-glucose
1.07	4.60	0	PS1
1.03	48.36	0	PS4
1.20	24.57	0	Sucrose
0.95	1.26	0	PS6
0.89	0.01	0	Formate
1.00	34.20	0	PC1
6.40	32.41	0	PC1sc
0.00	18.25	0	MICoV
0.11	11.81	0	mean

Table s4: Matera grain: Variogram fitting results

var	len_scale	nugget	Metabolite
0.92	0.00	0	Val
0.70	0.00	0	Ile
0.78	0.07	0	U1
0.76	0.02	0	U2
0.97	3.86	0	Ala
1415.08	458484.47	0	Acetic acid
0.98	1.94	0	Succinate
0.88	1.72	0	Asn
0.87	0.00	0	U5
1.00	6.59	0	U6
0.78	0.22	0	b-glucose
1.02	0.05	0	Melibiose
0.86	0.00	0	a-glucose
1.05	0.02	0	PS4
0.85	0.05	0	Sucrose
0.94	6.10	0	PS5
0.74	2.86	0	PS6
0.07	36.37	0	PS7
0.96	6.13	0	Fumarate
1.04	9.31	0	Trp
4124.33	501865.78	0	Formate
0.36	6.99	0	PC1
5.85	0.01	0	PC1sc
0.00	0.11	0	MICoV
0.27	0.01	0	mean

Average metabolite profiles

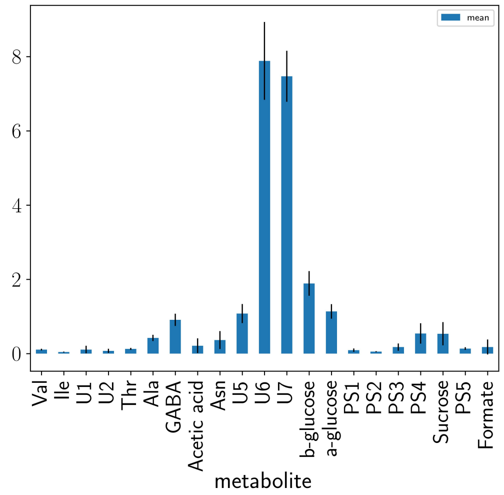

(a) Wheat shoots harvested in Genzano field.

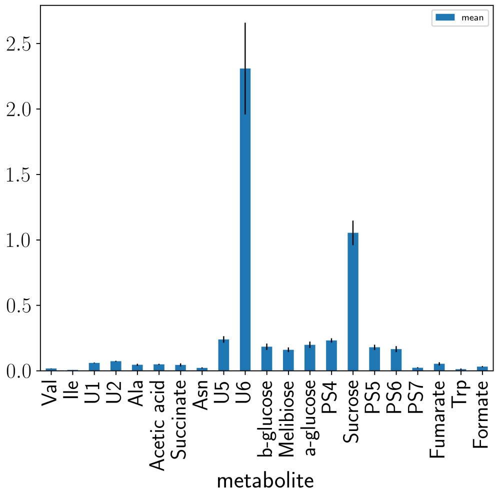

(b) Grain harvested in Genzano field.

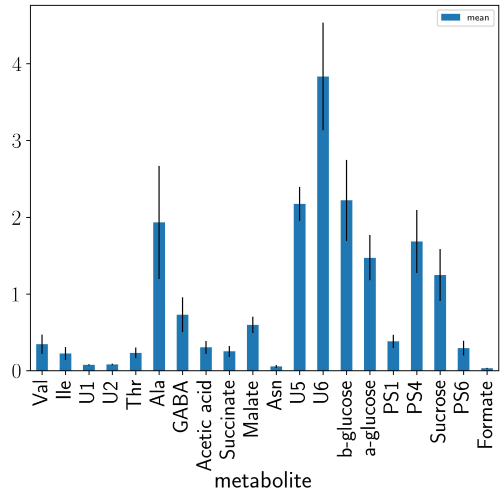

(c) Blooming spikes harvested in Matera field.

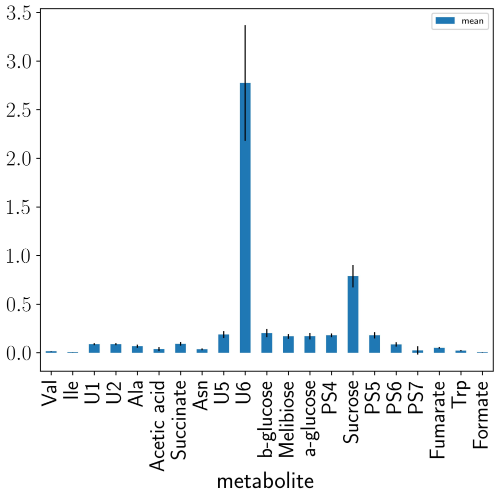

(d) Grain harvested in Matera field.

Figure s1: Mean value of each metabolite calculated over all points in each field together with the corresponding standard deviations (error bars).

Radial Bases Function interpolation of Acetic acid and PS3

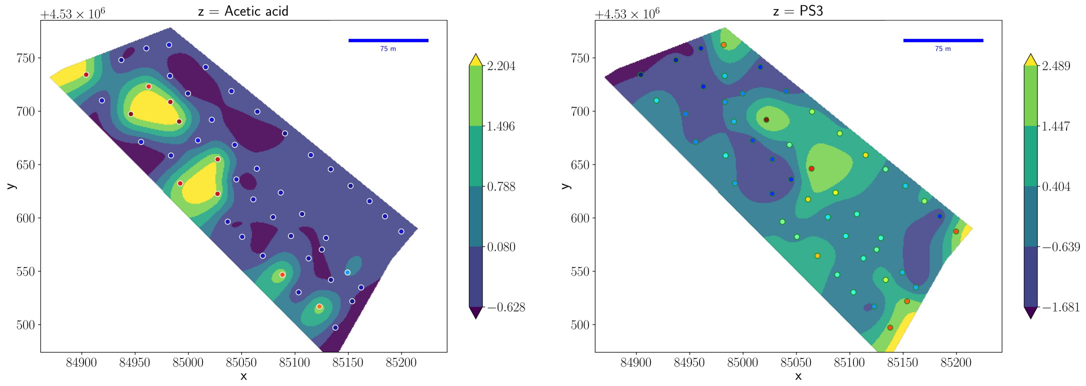

(a) Acetic acid.

(b) Unknown polysaccharide PS3.

Figure s2: Heat map of two significative metabolites, quantified from extracts of wheat shoots, interpolated by RBF.

Moran's Index

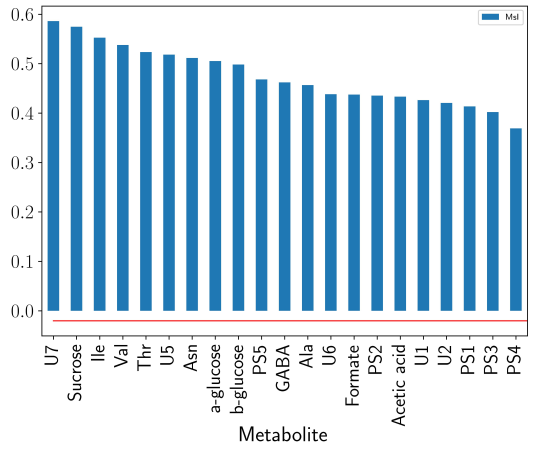

(a) Wheat shoots harvested in Genzano field.

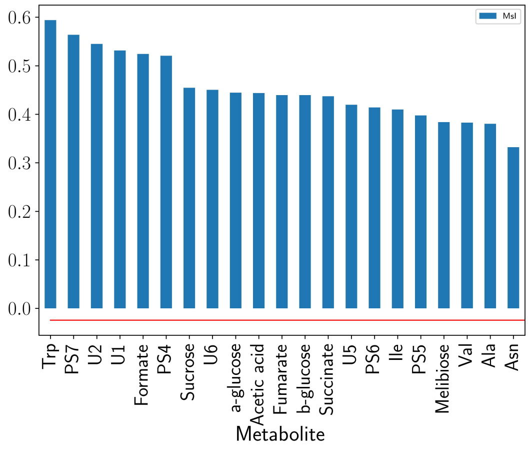

(b) Grain harvested in Genzano field.

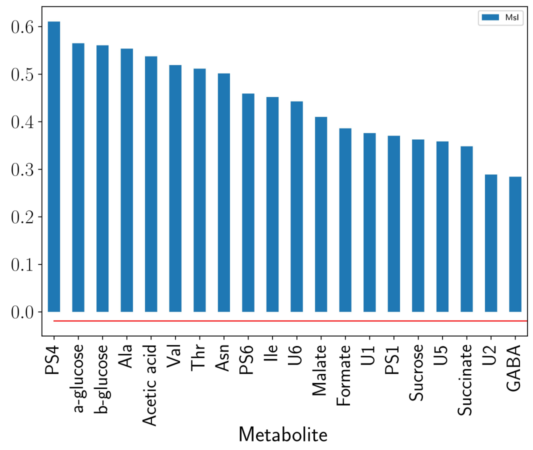

(c) Blooming spikes harvested in Matera field.

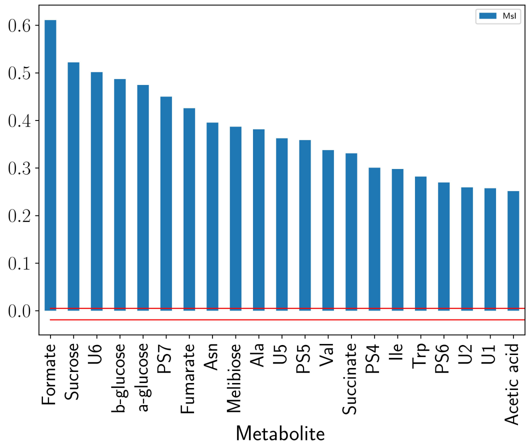

(d) Grain harvested in Matera field.

Figure s3: Moran's Index for each quatified metabolite.

RBF Interpolation of metabolic index based on the mean

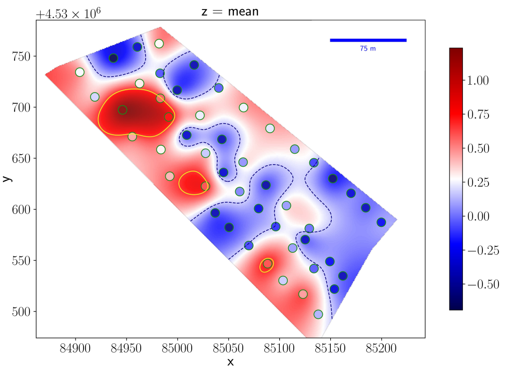

(a) Wheat shoots harvested in Genzano field.

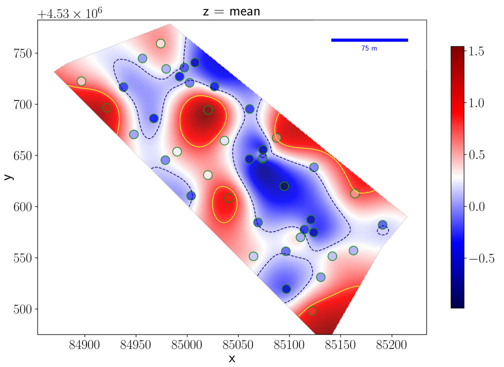

(b) Seeds harvested in Genzano field.

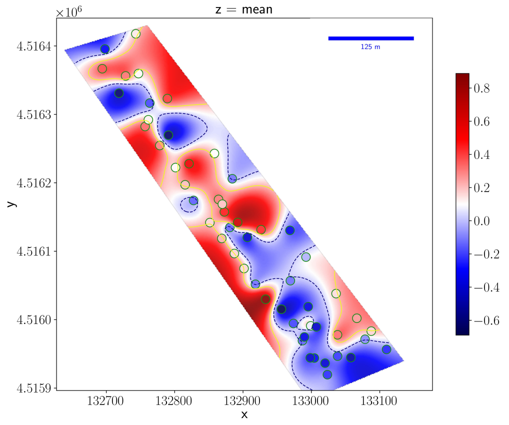

(c) Spikes harvested in Matera field.

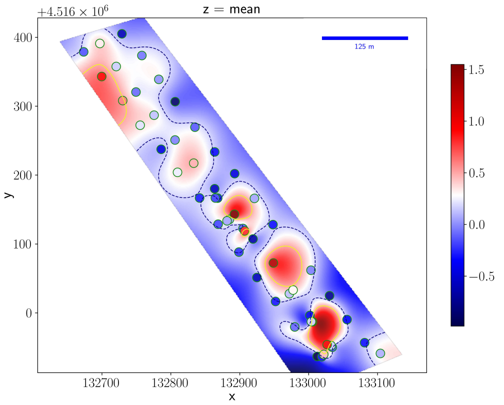

(d) Seeds harvested in Matera field.

Figure s4: Deterministic interpolation of MI_{mean} by Radial Basis Functions.

RBF Interpolation of metabolic index based on the coeffiecient of variation (CoV)

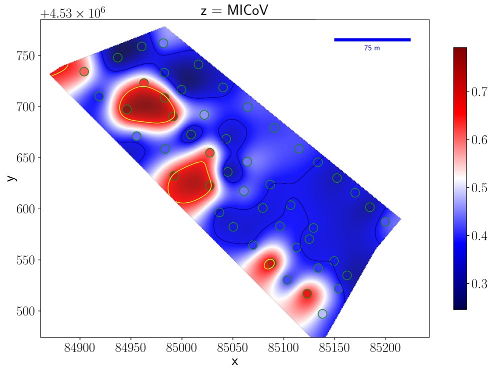

(a) Wheat shoots harvested in Genzano field.

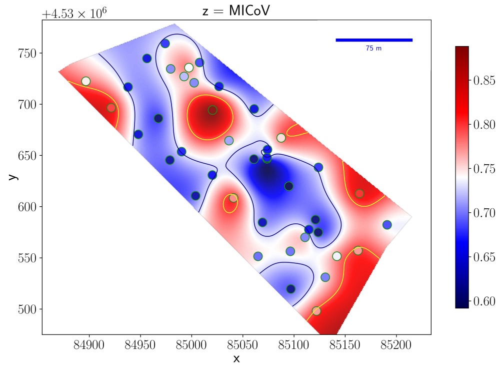

(b) Grain harvested in Genzano field.

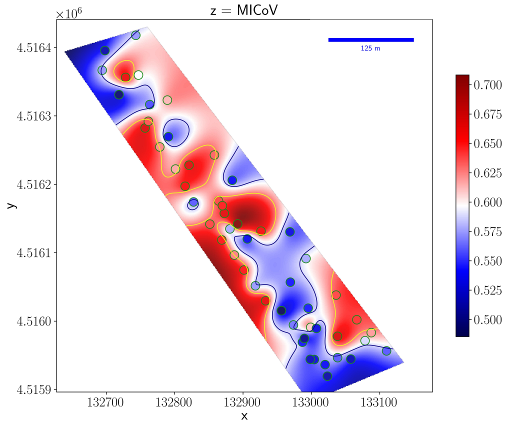

(c) Blooming spikes harvested in Matera field.

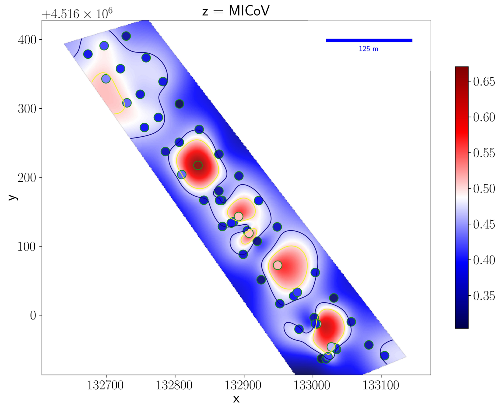

(d) Grain harvested in Matera field.

Figure s5: Deterministic interpolation of MI_{CoV} by Radial Basis Functions.

Local Indicators of Spatial Association (LISA) analysis

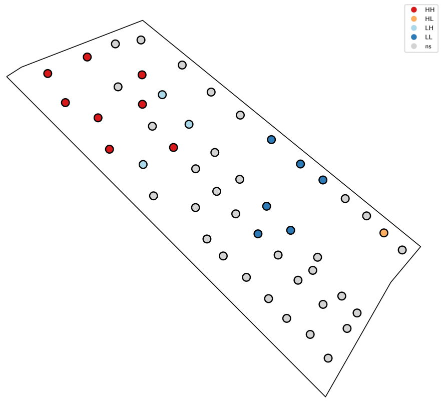

(a) Wheat shoots harvested in Genzano field.

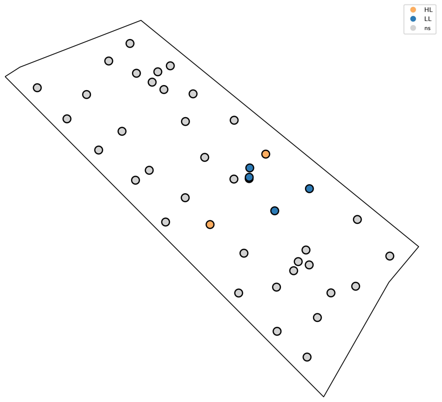

(b) Grain harvested in Genzano field.

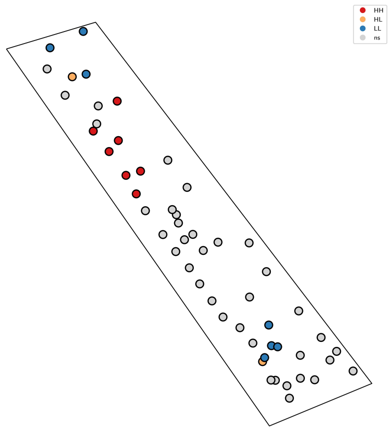

(c) Blooming spikes harvested in Matera field.

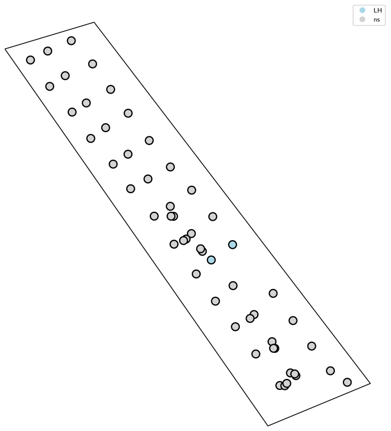

(d) Grain harvested in Matera field.

Figure s6: LISA maps of MI.
